# Semiautomatic intermittent pneumatic compression device applied to deep vein thrombosis in major orthopedic surgery

**DOI:** 10.1186/s12938-018-0513-5

**Published:** 2018-06-15

**Authors:** Dapeng Wang, Fuqin Bao, Qiang Li, Yugang Teng, Jianjun Li

**Affiliations:** 1 Department of Orthopedic Surgery, Shengjing Hospital of China Medical University, Shenyang, 11000 Liaoning China; 2 Department of Orthopedic Surgery, Fuxin Central Hospital, Fuxin, 12300 Liaoning China

**Keywords:** Orthopedic surgery, Deep vein thrombosis, Intermittent pneumatic compression device, Graduated compression stockings

## Abstract

**Objective:**

To investigate the effect of additional semiautomatic intermittent pneumatic compression device (IPCD) in the prevention of deep vein thrombosis (DVT) of lower extremity in these patients undergoing major orthopedic surgery, when compared with the conventional graduated compression stockings alone.

**Methods:**

The data of 112 patients undergoing major orthopedic surgery were retrospectively analyzed. 51 patients who ever received IPCD and graduated compression stockings during major orthopedic surgery were taken as the experimental group, and 61 patients who only received the conventional graduated compression stockings during surgery were taken as the observation group. The Doppler sonography was utilized to detect the presence of DVT and pulmonary embolism pre- and postoperatively. Besides, the mean and peak velocity of blood flow in femoral vein were recorded before and after surgery. And then, the comparisons between the two groups were made, respectively.

**Results:**

When compared with the conventional graduated compression stockings alone, the intraoperative application of IPCD and stockings contributed the significant reduction of DVT (3.92%, 2/51 versus 9.84%, 6/61, X^2^ = 5.632, P = 0.034). In terms of the mean and peak velocity of blood flow in femoral vein, the postoperative difference was higher in the observation group than those in the control group (149.56 ± 26.35 versus 130.15 ± 22.56 mm/s, P < 0.05). With respect to perioperative blood loss, the difference between the two groups was statistically significant (800.5 ± 320.7 versus 950.1 ± 305.9 ml, P = 0.031).

**Conclusions:**

Intraoperative application of IPCD could promote blood circulation of lower limbs, and significantly decrease the incidence of potentially fatal DVT in patients undergoing major orthopedic surgery, when compared with the conventional graduated compression stockings.

## Background

In general, the major orthopedic surgery includes total hip arthroplasty (THA), total knee arthroplasty (TKA), and hip fraction surgery (HFS), which are associated with significant morbidity and mortality, and are especially attributable to the high risk of postoperative venous thromboembolism, on account of excessive production of procoagulants, perioperative immobilization and cement polymerization, etc. [1–5]. During and after major orthopedic surgery, the risk of venous thromboembolism approximates 50–80%, and might persist for up 3 to 6 months after surgery [6]. According to the risk classification of venous thromboembolism in patients undergoing orthopedic surgery, these patients with major orthopaedic surgery are at high risk of deep vein thrombosis (DVT) [7, 8]. And, DVT refers to the abnormal coagulation of blood in the deep veins and obstruction of vein lumen, thereby leading to the blockage of venous return. Additionally, being regarded as a serious and even life-threatening complication, DVT mostly occurs in the lower extremities [9–11]. It can not only transform into chronic vascular diseases, affect life quality of patients, but also lead to fateful pulmonary embolism. In addition, on the basis of previous literatures, after the confirmation of venous angiography, the incidences of DVT after THA and TKA without preventive measures was 42% to 57% and 41% to 85%, respectively, and the incidence of DVT after HFS was 45–70% [12–14]. Therefore, when compared with other patients, it is more necessary to prevent DVT in patients who ever undergo major orthopedic surgeries [15, 16].

At present, the preventive measures for perioperative DVT in major orthopedic surgery mainly include drug and physical treatment. And, the commonly utilized physical measures are intermittent pneumatic compression device (IPCD), graduated compression stockings (GCS), and plantar vein pumps, etc. These physical treatment can be used alone or combined with drugs. Although the anticoagulant drugs including heparin, coumarins, and fondaparinux, can effectively prevent DVT of lower extremities, it can also increase the bleeding risk. It is not suitable for patients with high bleeding risk. Once the severe bleeding occurs, it is often more intractable than DVT in the lower extremities. In addition, the critically ill patients are generally in critical condition and have relatively high bleeding risk, so they are not suitable for general anticoagulant therapy. The common physical measures include IPCD, gradient elastic stockings, plantar vein pumps, etc. Currently, they can be used alone or as an auxiliary measure for drug prevention, and are an important strategies for DVT prevention [17]. Specifically, IPCD can be considered as one common mechanical precaution for DVT in clinical practice [18, 19]. As far as we are concerned, IPCD is a engineering device that uses external mechanical force to compress the veins of lower extremities and promote blood return, which can effectively prevent the occurrence of DVT [20]. In this research, the authors aimed to investigate the therapeutic effect of intermittent pneumatic compression device in preventing DVT of lower extremity in these patients who ever underwent major orthopedic surgery, and we reported the detailed information as followed.

### Patients

From March 2016 to February 2018, 112 patients who ever underwent major orthopedic surgeries were eligible. The patients who had DVT in lower extremities, life-threatening patients, and patients with APACHE (Acute Physiology and Chronic Health) score ≧ 20 points, the patients suffering from lower extremity lesions could not received IPCD or graduated compression stockings were excluded. Besides, the other exclusion criteria are as follows. (1) age < 18 years; (2) abnormal blood coagulation; (3) combines with heart function ≤ Level 3; (4) chronic renal failure ≥ 3 phase; (5) severe peripheral arterial disease; (6) Acute thrombophlebitis; (7) Neurological diseases of lower limbs; (8) Joint stiffness of lower limbs; (9) Recently received anticoagulant therapy; (10) History of bleeding disorders or malignancy; (11) Allergic to heparin. (12) unavailability of either pre- or postoperative Doppler sonography. On account of lack of pre- or postoperative Doppler sonography, 24 patients were excluded.

After rigorous process of selection, the data of 112 patients undergoing major orthopedic surgery were retrospectively analyzed. 51 patients who ever received IPCD and stockings during major orthopedic surgery were taken as the experimental group, and 61 patients who only received the conventional graduated compression stockings during surgery were taken as the observation group. In the experimental group, there were 29 males and 22 females, the age ranged from 38 to 79 years old, and the average age was 55.6 ± 14.2 years old. 18 cases underwent hip replacement, 12 cases underwent knee replacement, 21 cases underwent fracture surgical treatment. In the control group, there were 36 males and 25 females, the age ranged from 35 to 81 years old, and the average age was 50.6 ± 19.2 years old. 22 cases underwent hip replacement, 15 cases underwent knee replacement, 24 cases underwent fracture surgical treatment. The differences in gender composition, age distribution, and types of diseases between the two groups were not statistically significant (P > 0.05). The baseline characteristics are displayed in Table 1.

**Table 1:** Baseline characteristics of included patients

	Experimental group (N = 51)	Control group (N = 61)
Gender (male/female)	29/22	36/25
Age (x ± s, years old)	55.6 ± 14.2	50.6 ± 19.2
Body weight (x ± s, Kg)	61.2 ± 9.5	61.8 ± 8.5
Primary diseases		
Femoral neck fracture	14	15
Ischemic necrosis of femoral head	6	9
Hip osteoarthritis	7	9
Knee joint osteoarthritis	11	15
Femoral dysplasia	13	13
Anesthesia method		
General anesthesia in the trachea	45	51
Continuous epidural anesthesia	6	10
Prosthesis fixation		
Non-cement fixation	7	9
Cement fixation	32	36
Mixed fixation	12	16

## Methods

All these selected patients received routinely anticoagulant therapy via intraperitoneal injection (Fraxiparine, 0.4 ml, GlaxoSmithKline) at 12 h before surgery or 10 h after surgery, and then repeated every 24 h until 10 days after surgery. In the experimental group, the patients received IPCD (SCDTM system, Kendal, America) and additional stockings during major orthopedic surgery. And the detailed procedures are described as follows. First of all, the patients were required to wear the graduated compression stockings before operation. Secondly, the patients started to receive IPCD treatment in the non-surgical side once the anesthesia beginned, and then receive bilateral IPCD treatment after operation. On the first postoperative day, continuous use was required for 24 h. Afterwards, the patients could use it several times one day, but should ensure that the total daily use time is 6–8 h. All these patients used IPC until 10 days after surgery, regardless of whether they could be able to get out of bed. By means of a standard air pulse generator, the standard thromboprophylaxis mode was maintained throughout the complete procedure until extubation. And, these were intermittently inflated to a maximum pressure of 45 mm Hg. In particular, the sequential compression which is used once every minute refers to inflate distal chambers fist, then continue to the more proximal chambers in a wavelike.

**Fig. 1:**
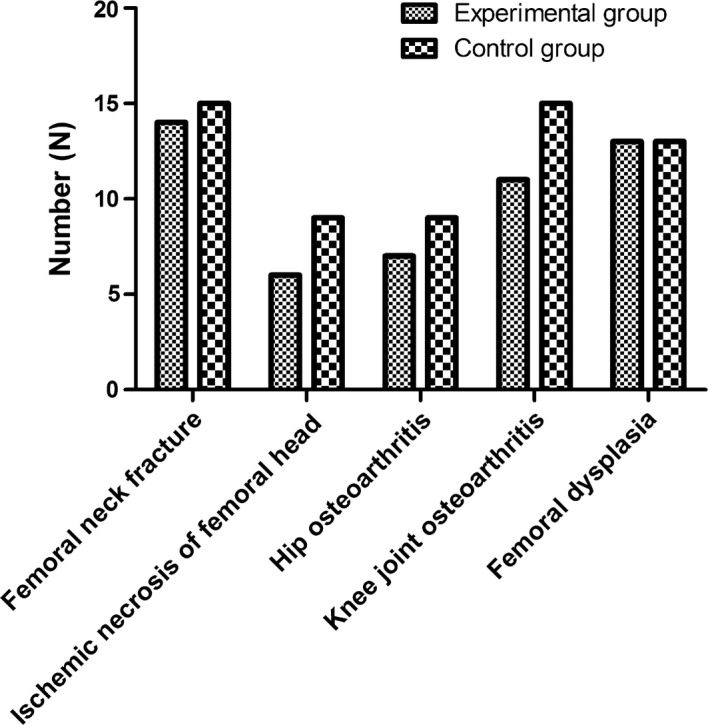
Comparison of primary diseases between graduated compression stockings alone and IPCD in addition

**Fig. 2:**
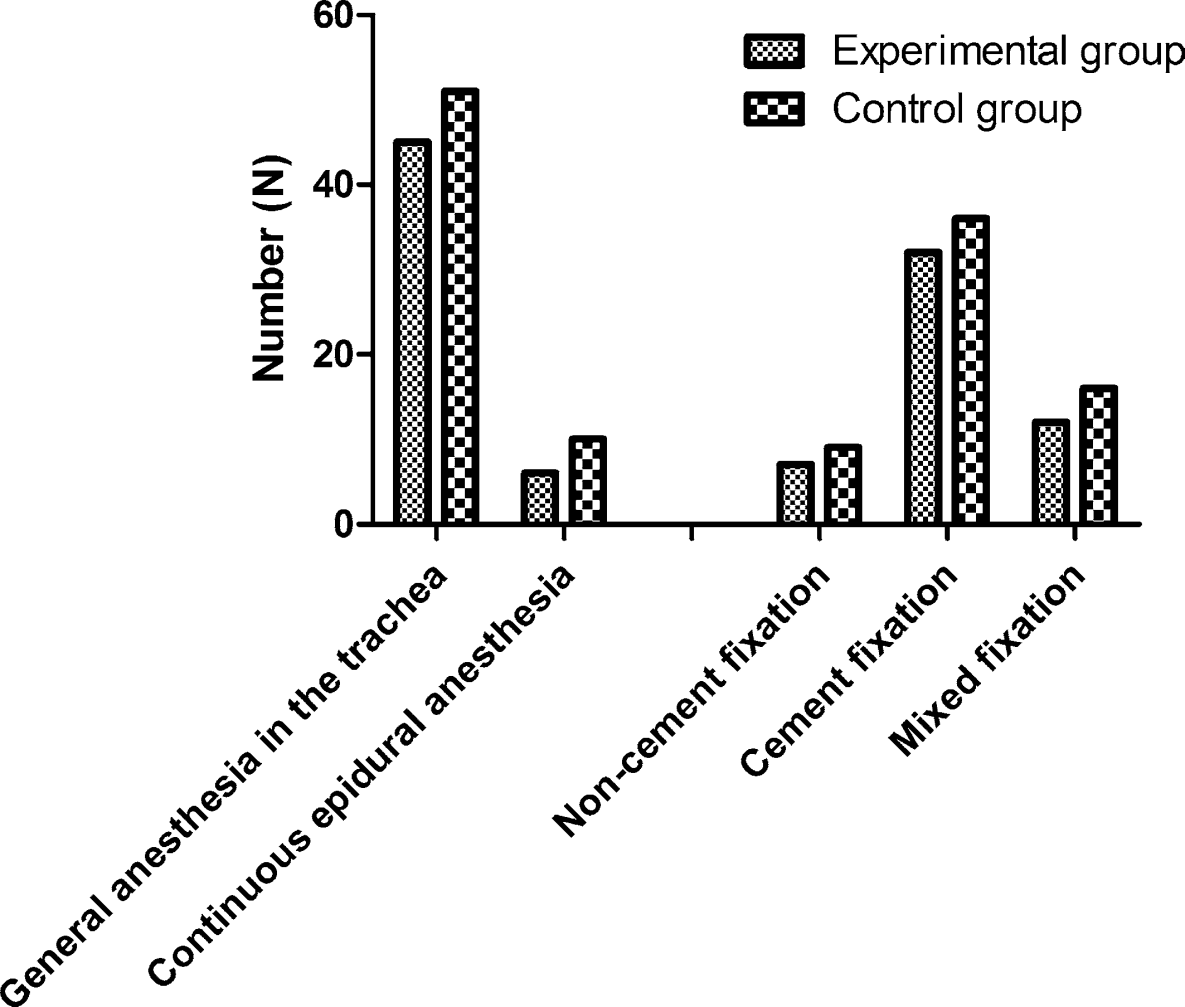
Comparison of anesthesia method and prosthesis fixation between graduated compression stockings alone and IPCD in addition

The Doppler sonography (Sonoline Antares, Siemens Sector Healthcare) was utilized to detect the presence of DVT and pulmonary embolism pre- and postoperatively, and this performance was carried out by the same examiner who has the professional certification and was blinded to the group status of all patients. Besides, the mean and peak velocity of blood flow in femoral vein, and blood flow volume were recorded before the surgery and on the 2th, 5th and 7th day after surgery. And then, the comparisons between the two groups were made, respectively. In addition, the intraoperative blood loss and postoperative wound drainage were recorded, and the bleeding signs were observed within 4 weeks after the operation, such as fetal hemorrhage, hemoptysis, hematuria, cerebral hemorrhage, etc. (Figs. 1, 2).

### Statistical analysis

Statistical Package for the Social Sciences (SPSS) version 6.0 was used for the analyses. To analyze the differences between the specialties, we used cross tabs with Chi square test and correlation was calculated with the Spearman’s rho. The alpha level was set at 0.05.

## Results

### Comparison of DVT incidence between graduated compression stockings alone and IPCD in addition

**Fig. 3:**
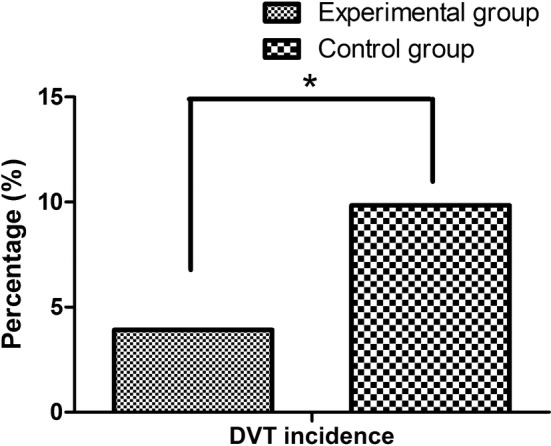
Comparison of DVT incidence between graduated compression stockings alone and IPCD in addition

In the control group, 6 of 61 patients (9.84%) developed DVT by the postoperative Doppler sonography. On the contrary, only 2 of 51 patients (3.92%) in the experimental group developed DVT. The difference in DVT incidence between both groups as determined by Fisher’s test was statistically significant (X^2^ = 5.632, P = 0.034). When compared with the conventional graduated compression stockings alone, the intraoperative application of IPCD and stockings contributed the significant reduction of DVT (3.92%, 2/51 versus 9.84%, 6/61, *X*^2^ = 5.632, P = 0.034), as shown in Fig. 3.

### Comparison of hemodynamic parameters between graduated compression stockings alone and IPCD in addition

**Fig. 4:**
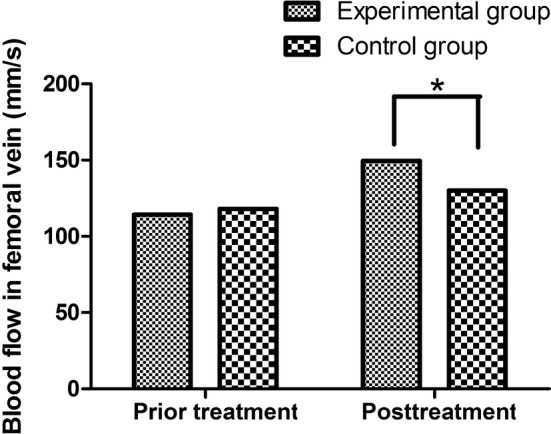
Comparison of blood flow in femoral vein between graduated compression stockings alone and IPCD in addition

In terms of the mean and peak velocity of blood flow in femoral vein, the postoperative difference was higher in the observation group than those in the control group (P < 0.05). As shown in Table 2 and Fig. 4, the perioperative blood flow of femoral vein in graduated compression stockings alone was 130.15 ± 22.56 mm/s, and the perioperative blood flow of femoral vein in graduated compression stockings plus IPCD group was 149.56 ± 26.35 mm/s. Prior to treatment, the difference between the two groups was not statistically significant (114.23 ± 23.69 versus 118.12 ± 22.11 mm/s, P > 0.05).

**Table 2:** Comparison of blood flow in femoral vein between graduated compression stockings alone and IPCD in addition

Group	Number	Prior treatment	Posttreatment
Experimental group	51	114.23 ± 23.69	149.56 ± 26.35
Control group	61	118.12 ± 22.11	130.15 ± 22.56
t		0.017	12.011
P		0.954	0.000

### Comparison of perioperative blood loss and bleeding signs between graduated compression stockings alone and IPCD in addition

**Fig. 5:**
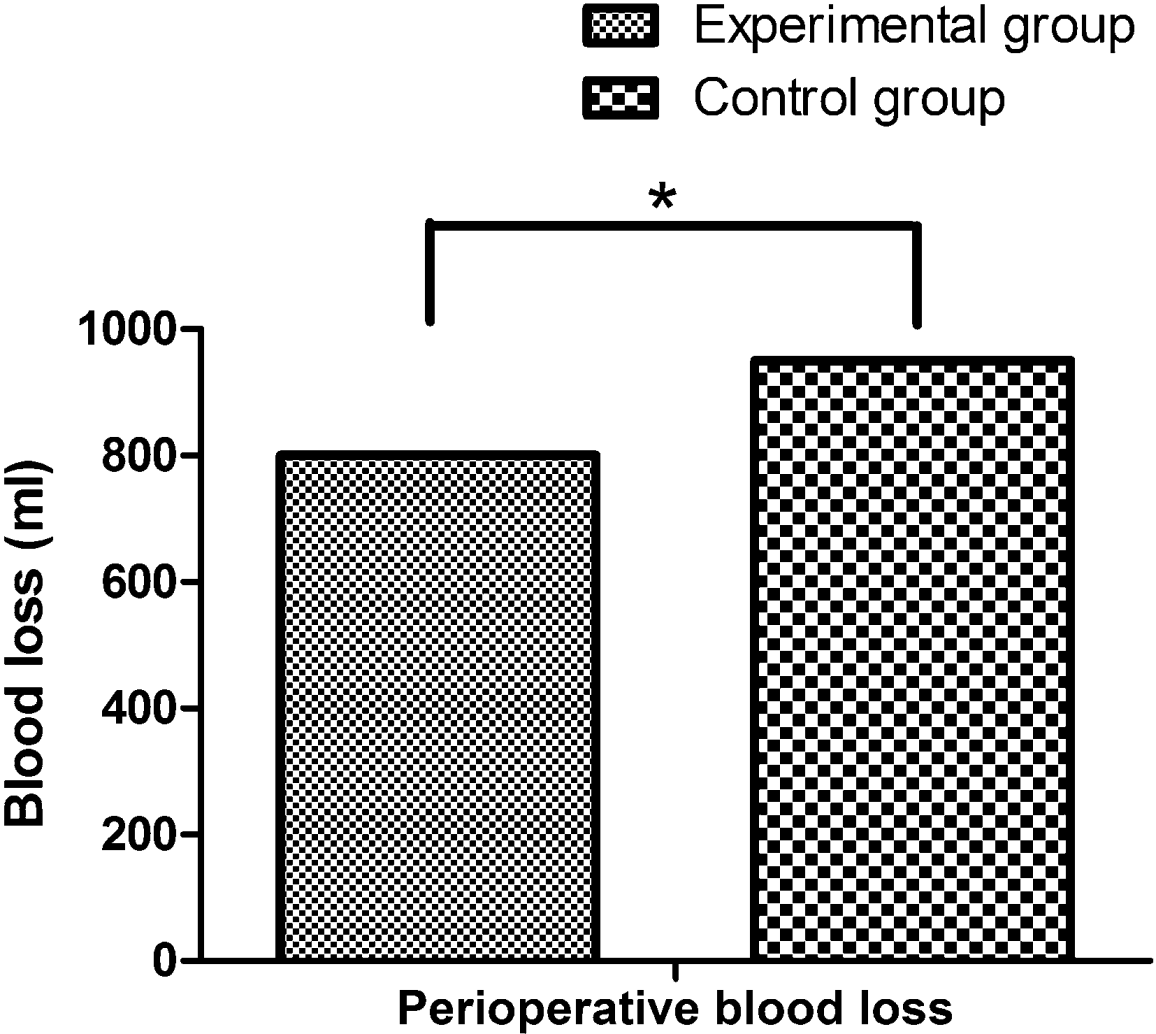
Comparison of perioperative blood loss and bleeding signs between graduated compression stockings alone and IPCD in addition

With respect to perioperative blood loss, as shown in Table 3 and Fig. 5, the perioperative blood loss in graduated compression stockings alone was 950.1 ± 305.9 ml, and the perioperative blood loss in graduated compression stockings plus IPCD group was 800.5 ± 320.7 ml. After statistical analysis, the difference between the two groups was statistically significant (P = 0.031). Besides, concerning the bleeding signs, only one patient in the control group suffered from postoperative upper gastrointestinal hemorrhage.

**Table 3:** Perioperative blood loss and bleeding signs between graduated compression stockings alone and IPCD in addition

Group	Number	Bleeding signs	Perioperative blood loss (ml)
Experimental group	51	0	800.5 ± 320.7
Control group	61	1	950.1 ± 305.9

## Discussion

In general, the slow blood flow, injured venous wall and blood hypercoagulability have been recognized as the three major factors leading to venous thromboembolism [21–23]. In clinical practice, these patients underwent major orthopedic surgery are routinely confined to bed rest for a long time, the blood flow becomes slow, and venous thromboembolisms are easy to form [24]. Generally speaking, the major orthopedic surgery can result in the high risk for venous thromboembolism, which can potentially lead to various chronic complications. Therefore, the DVT prophylaxis has been the conventional care for patients who ever undergo major orthopedic surgery in clinical practice [25, 26]. At present, a variety of treatment to prevent DVT are necessary and available in clinical practice, and the two primary approaches are pharmacological strategy and mechanical devices, such as antiplatelet, anticoagulant, IPCD, graduated compression stockings, and plantar vein pumps, etc. [27, 28]. Although the pharmacological strategy play an significant role in the prevention of DVT, it also has important potential risk of bleeding, prosthetic joint infections, and reoperation [28, 29]. According to previous publications, these increased risks after major orthopedic surgery has been widely documented in general population. And, all these above complications might cause increased hospital length of stay and costs, higher morbidities and death, and permanent removal of the prosthetic joint [30, 31]. Hence, the mechanical devices are thought to be inferior to pharmacological agents to prevent DVT [32].

In healthy populations, the venous pressure in the lower extremities is increasing from top to bottom. On the basis of this principle, the graduated compression stocking is one kind of elastic stocking that promotes venous return of the lower extremities. The basic mechanisms of DVT prevention by graduated compression stocking are as follows. Firstly, it can provide continuous pressure protection. The persistent and moderate venous wall pressure can offset the increased venous pressure caused by various causes [33]. The overly dilated veins of lower extremities are reduced, and the vein stasis can be improved, in order to accelerate the blood flow of lower extremities, prevent the aggregation of clotting factors and adhesion to the vascular intima, and promote the return of venous blood. Secondly, it can also provide the instant supercharging. Through the gradient pressure from bottom to top, the contraction of lower extremity muscle can produce an immediate and antidromic pressure wave which acts on the deep veins [5]. And this pressure can instantaneously increase the blood flow velocity. Such repeated contraction of muscles can continue to produce the antidromic compression force, which promotes venous return, maintains pulsation and circulation, and thereby effectively prevent DVT. Nevertheless, the following patients should not use elastic socks. (1) Suspect or confirm peripheral vascular disease; (2) Peripheral nerve or other sensory impairments; (3) Skin diseases, such as dermatitis, gangrene; (4) Allergy to elastic stockings; (5) Severe leg edema or pulmonary edema caused by heart failure, congestive heart failure; (6) Lower limb deformity. In addition, with respect to the pressure selection, the domestic and international guidelines are consistent with each other. The domestic guidelines for perioperative thromboprophylaxis in general surgery recommend that the ankle pressure should be maintained at 18–23 (1 mmHg = 0.133 kPa). And, the recommendation of international guideline is that the optimal pressures from ankle to middle thigh are 18, 14, 10, and 8 mmHg, respectively. The patients should constantly focus on whether the elastic stockings are worn correctly, because the actual pressure value is closely related to the correct wearing. In further, according to the guideline of health promotion in UK national institute, the patients should start to use elastic stockings until they have complete activity ability [5, 17, 33]. On the other hand, IPCD is a engineering device that can provide progressive pressure on the ankle, calf, and thigh. It also promotes venous blood flow of lower extremities and prevents DVT in this locations. The basic mechanisms of DVT prevention by IPCD are as follows. Firstly, it can accelerate the venous blood flow of lower extremities and promote the emptying of blood stasis. IPCD periodically pressurizes and decompresses the lower limbs, and thereby generates pulsatile blood flow which easily enters into the deep venous system of distal limb [34]. Hence, it can help to promote blood circulation in the lower extremities, to prevent coagulation factor aggregation and adhesion to the vascular intima, and thereby to prevent DVT. Secondly, it can increase the activity of fibrinolytic system. Not only for healthy population, but also for patients suffering from venous thrombosis, IPCD can help to stimulates endogenous fibrinolytic activity [35]. The use taboos of IPCD and graduated compression stockings are similar, and the patients suffering from deep venous thrombosis, thrombophlebitis, or pulmonary embolism should not received IPCD treatment [36]. This device can pressurize in a short time to accelerate venous blood flow of lower limbs, and the different pressure parameters can be adjusted. Usually, the pressures at ankle, calf and thigh are 45, 35 and 30 mmHg, respectively. Concerning the usage time, the guideline of British national health promotion institute recommends that the patients should start to use IPCD from hospital admission, until they have complete activity ability [37]. And the American Association of Chest Physicians recommends that the daily usage time should be more than 18 h. Although the physical prevention of deep venous thrombosis is the preferred method for patients with low, medium, and high risk of bleeding, it is still confronted with dire challenges, especially the patient compliance. And the measures which might improve compliance should be recommended, for instance, increase the equipment number, carry out professional training, increase the visit number by nurses, change health education methods and timing, improve or innovate device designs, strengthen nurses’ awareness of deep vein thrombosis prevention, etc. [18, 38].

In this research, concerning the comparison of DVT incidence between two groups, when compared with the conventional graduated compression stockings alone, the intraoperative application of IPCD and stockings contributed the significant reduction of DVT (3.92%, 2/51 versus 9.84%, 6/61, X2 = 5.632, P = 0.034). In terms of the mean and peak velocity of blood flow in femoral vein, the postoperative difference was higher in the observation group than those in the control group (P < 0.05). With respect to perioperative blood loss, the perioperative blood loss in graduated compression stockings alone was 950.1 ± 305.9 ml, and the perioperative blood loss in graduated compression stockings plus IPCD group was 800.5 ± 320.7 ml. After statistical analysis, the difference between the two groups was statistically significant (P = 0.031). Of course, several limitations should be acknowledged in this study, Firstly, the number of patients included in the study was relatively small and the pathological types were relatively simplex. Secondly, the optimal treatment time and period were not evaluated. Therefore, further multi-center studies with large samples are needed urgently.

## Conclusions

In summary, the intraoperative application of IPCD could promote blood circulation of lower limbs, and significantly decrease the incidence of potentially fatal DVT in patients undergoing major orthopedic surgery, when compared with the conventional graduated compression stockings alone.
